# A Heterologous Challenge Rescues the Attenuated Immunogenicity of SARS-CoV-2 Omicron BA.1 Variant in Syrian Hamster Model

**DOI:** 10.1128/jvi.01684-22

**Published:** 2023-01-18

**Authors:** Jian Ma, Xuan Liu, Ming Zhou, Peiwen Chen, Rirong Chen, Jia Wang, Huachen Zhu, Kun Wu, Jianghui Ye, Yali Zhang, Quan Yuan, Qiyi Tang, Lunzhi Yuan, Tong Cheng, Yi Guan, Ningshao Xia

**Affiliations:** a State Key Laboratory of Molecular Vaccinology and Molecular Diagnostics, National Institute of Diagnostics and Vaccine Development in Infectious Diseases, School of Life Sciences, School of Public Health, Xiamen University, Xiamen, Fujian, China; b State Key Laboratory of Emerging Infectious Diseases, School of Public Health, Li Ka Shing Faculty of Medicine, University of Hong Kong, Hong Kong SAR, China; c Guangdong-Hong Kong Joint Laboratory of Emerging Infectious Diseases/Joint Laboratory for International Collaboration in Virology and Emerging Infectious Diseases, Joint Institute of Virology (STU/HKU), Shantou University, Shantou, Guangdong, China; d EKIH Pathogen Research Institute, Futian District, Shenzhen, Guangdong, China; e Department of Microbiology, Howard University College of Medicine, Washington, DC, USA; University of North Carolina at Chapel Hill

**Keywords:** SARS-CoV-2, Omicron, immunogenicity, homologous and heterologous rechallenge, cross-variant neutralization

## Abstract

The severe acute respiratory syndrome coronavirus 2 (SARS-CoV-2) Omicron variant is becoming a dominant circulator and has several mutations in the spike glycoprotein, which may cause shifts of immunogenicity, so as to result in immune escape and breakthrough infection among the already infected or vaccinated populations. It is unclear whether infection with Omicron could generate adequate cross-variant protection. To investigate this possibility, we used Syrian hamsters as an animal model for infection of SARS-CoV-2. The serum from Omicron BA.1 variant-infected hamsters showed a significantly lower neutralization effect against infection of the same or different SARS-CoV-2 variants than the serum from Beta variant-infected hamsters. Furthermore, the serum from Omicron BA.1 variant-infected hamsters were insufficient to protect against rechallenge of SARS-CoV-2 Prototype, Beta and Delta variants and itself. Importantly, we found that rechallenge with different SARS-CoV-2 lineages elevated cross-variant serum neutralization titers. Overall, our findings indicate a weakened immunogenicity feature of Omicron BA.1 variant that can be overcome by rechallenge of a different SARS-CoV-2 lineages. Our results may lead to a new guideline in generation and use of the vaccinations to combat the pandemic of SARS-CoV-2 Omicron variant and possible new variants.

**IMPORTANCE** The severe acute respiratory syndrome coronavirus 2 (SARS-CoV-2) Omicron variant causes breakthrough infections among convalescent patients and vaccinated populations. However, Omicron does not generate robust cross-protective responses. Here, we investigate whether heterologous SARS-CoV-2 challenge is able to enhance antibody response in a sensitive animal model, namely, Syrian hamster. Of note, a heterologous challenge of Beta and Omicron BA.1 variant significantly broadens the breadth of SARS-CoV-2 neutralizing responses against the prototype, Beta, Delta, and Omicron BA.1 variants. Our findings confirm that vaccination strategy with heterologous antigens might be a good option to protect against the evolving SARS-CoV-2.

## INTRODUCTION

The pandemic of severe acute respiratory syndrome coronavirus 2 (SARS-CoV-2) led to more than 600 million infection cases and over 6 million confirmed deaths worldwide in the past 2 years. SARS-CoV-2 infects host cells via a specific binding of the spike glycoprotein with angiotensin-converting enzyme 2 (ACE2) receptor (1), which determines the viral tissue tropism. Respiratory tract is the primary target organ of SARS-CoV-2, causing its airborne transmission (2). SARS-CoV-2-infected individuals may develop coronavirus disease 2019 (COVID-19), which presents different symptoms with varied severities such as cough, fever, pneumonia, and multiorgan failure seen in approximately 20% of hospitalized patients (2). Asymptomatic people may carry and shed virus for transmission. Unfortunately, persistent symptoms and sequelae are usually observed in the discharged patients (3–5) and are also demonstrated in experimental animals that recovered from COVID-19 (6). Currently approved vaccines are playing important roles in mitigating the ongoing pandemic and reducing SARS-CoV-2-caused deaths. However, the protection efficiency of SARS-CoV-2 vaccines are challenging by the new variants.

In order to evade human immune responses, SARS-CoV-2 mutates frequently, resulting in numerous circulating variants (7). The emerging and re-emerging of SARS-CoV-2 variants pose a critical threat to global public health. These variants gather dramatic immunogenicity changes through critical mutations in the spike glycoprotein, which usually cause immune escape from the neutralizing antibodies elicited by previous infection and vaccination (8). For instance, the variant lineages of Beta (B.1.351), Delta (B.1.617.2), and Omicron (B.1.1529) obtain significantly elevated resistance to vaccines and neutralizing antibodies through several critical mutations such as E484K, N501Y, L452R, T478K, and Q498R, etc. (9–12). Since December 2021, the Omicron variant has become the globally dominant circulating strain and causes millions of breakthrough infections among the previously exposed and vaccinated populations (13–15). However, the distinct immunogenicity features of Omicron strain are not fully comprehended. We wonder whether Omicron variant infection can generate sufficient immunity against rechallenge of itself or other variant lineages. In this study, we investigated the immunogenicity features of Omicron BA.1 variant and cross-variant protection efficiency of Omicron infection in a Syrian hamster model and found that SARS-CoV-2 Omicron BA.1 variant infection elicits antibodies with a weak neutralization effect that can be strengthened by reinfection of a different lineages of SARS-CoV-2.

## RESULTS

### Omicron BA.1 variant infection resulted in weak protection against rechallenges of the same or different SARS-CoV-2.

**FIG 1:**
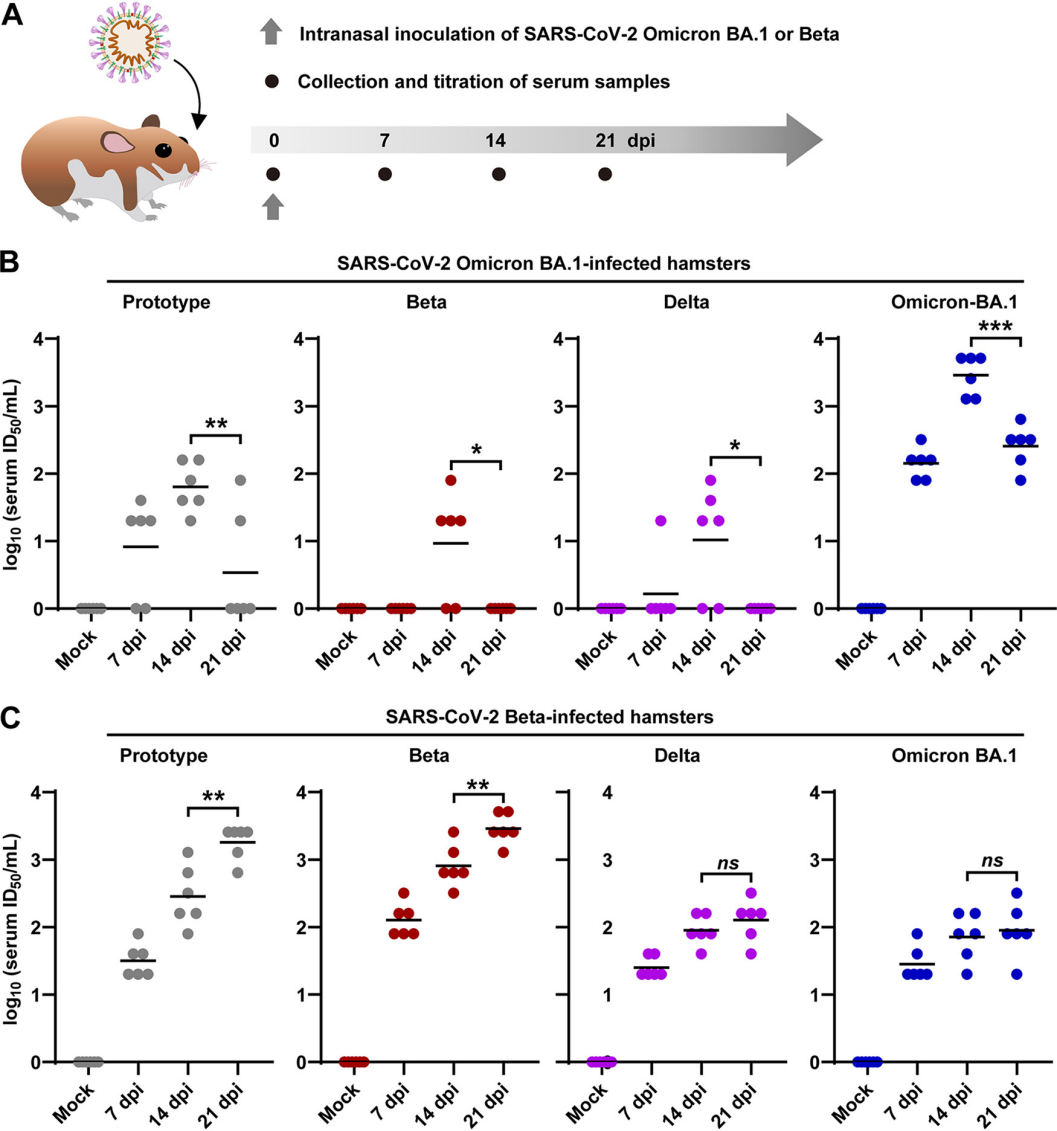
Inhibitory effects of the serum from Omicron BA.1 or Beta variant-infected hamsters on the infection-caused cytopathic effect (CPE) of different variants. (A) Scheme of severe acute respiratory syndrome coronavirus 2 (SARS-CoV-2) infection and serum sample collection in a hamster model. (B, C) The serum samples were collected from hamsters previously exposed to 1 × 10^3^ PFU of SARS-CoV-2 Omicron BA.1 (B) or Beta (C) variants at 0, 7, 14, and 21 days post infection (dpi), respectively (*n* = 6/group). The variant-specific serum neutralization titers against SARS-CoV-2 prototype, Beta, Delta, and Omicron BA.1 variants were measured by a titration method based on a titration method of 50% tissue culture infective dose (TCID_50_) inhibition. ID_50_, 50% infective dose; ns, not significant; *, *P* < 0.05; **, *P* < 0.01; ***, *P* < 0.001.

Wishing to know whether the antibodies against SARS-CoV-2 Omicron variant can provide adequate protection against infection of SARS-CoV-2 prototype and other variants, we infected Syrian hamsters with 1 × 10^3^ plaque forming unit (PFU) of Omicron BA.1 variant by intranasal route as previously described (16–18), and collected serum samples at 0, 7, 14, and 21 days postinfection (dpi), respectively (Fig. 1A). The hamsters infected with 1 × 10^3^ PFU of Beta variant were set as controls. The cross-variant serum neutralization titers against SARS-CoV-2 prototype, Beta, Delta, and Omicron variants were measured by examining the ability to inhibit virus-caused cytopathic effect (CPE) using a titration assay (50% tissue culture infective dose [TCID_50_]) in Vero cells. First, our neutralization assay showed that the serum from Omicron BA.1-infected hamsters reached a peak neutralization titer at 14 dpi (Fig. 1B), while those from Beta-infected hamsters presented increasing neutralization titer (Fig. 1C). Second, serum from Omicron BA.1-infected hamsters had a nearly 100-fold stronger neutralization against Omicron BA.1 virus than against other variants, including prototype, Beta, or Delta (Fig. 1B) at 14 or 21 dpi. Third, the serum from Beta-infected hamsters showed higher neutralization titers against the prototype, Beta, and Delta variant than those from Omicron BA.1-infected hamsters. Moreover, the serum from Beta-infected hamsters also presented a better neutralization effect against itself than against prototype, Delta, and Omicron BA.1 variants at 7, 14, and 21 dpi (Fig. 1C). Therefore, our initial experiments suggested that Omicron BA.1 infection might cause a weaker immunization effect against rechallenge of itself and other SARS-CoV-2 variant lineages.

**FIG 2:**
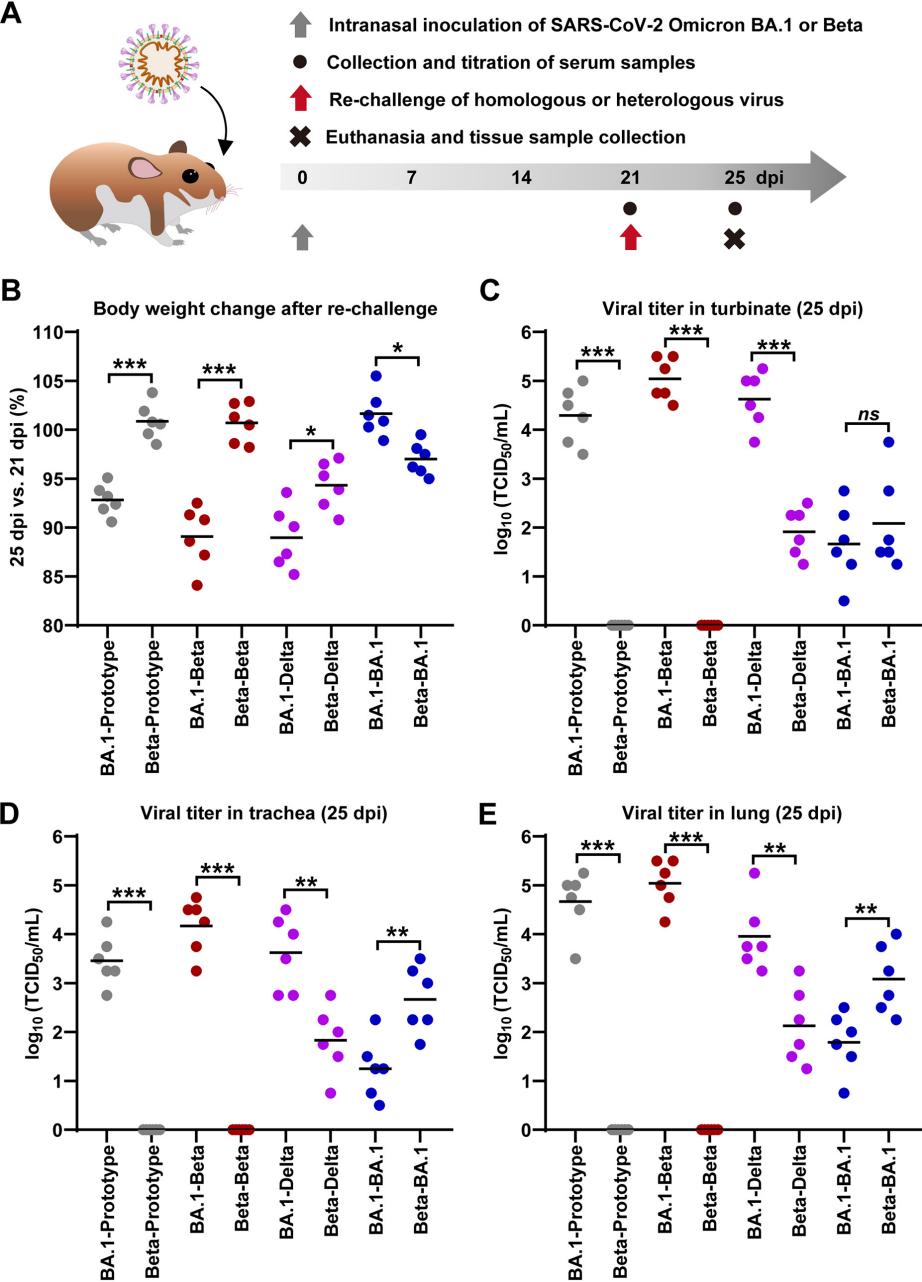
Rechallenge of SARS-CoV-2 in hamsters previously exposed to Omicron BA.1 and Beta variants. (A) The experimental scheme of the infection and rechallenge of 1 × 10^3^ PFU of SARS-CoV-2 Omicron or Beta variants in hamsters. The infected animals were reinfected with 1 × 10^3^ PFU of SARS-CoV-2 prototype, Beta, Delta, and Omicron variants at 21 dpi, respectively (*n* = 6/group). All of the hamsters were euthanized at 25 dpi (4 days after rechallenge) for the desired analysis. (B) Body weight changes from 21 to 25 dpi were recorded. (C to E) Viral titers in the tissues collected from respiratory tract organs, including turbinate (C), trachea (D), and lung (E) were measured by a titration method of TCID_50_. Because the detection limitation of titration method is at least 10 TCID_50_/mL, the undetectable samples are shown as “0” in the figures.

Afterward, we attempted to make clear whether the primary Omicron BA.1 infection could protect animals against challenges of itself and other variant lineages. In doing so, the hamsters were initially infected with 1 × 10^3^ PFU of SARS-CoV-2 Beta or Omicron BA.1 variants and were then rechallenged with 1 × 10^3^ PFU of SARS-CoV-2 prototype, Beta, Delta, or Omicron BA.1 by intranasal route at 21 dpi (Fig. 2A). These hamsters were euthanized at 25 dpi (4 days after rechallenge). Serum samples were collected at 21 and 25 dpi, and tissues of respiratory tract organs, including turbinate, trachea, and lung, were collected at 25 dpi for the desired experiments. First, we examined the body weights of the animals at 21 dpi before the rechallenge and at 25 dpi when it is 4 days after rechallenge. The body weight was shown as a ratio by comparing the average body weight at 25 dpi to that at 21 dpi. As shown in Fig. 2B, for the hamsters initially infected with Omicron BA.1 variant, rechallenges of SARS-CoV-2 prototype, Beta, and Delta caused 5% to 10% of body weight loss within 4 days. For the hamsters initially infected with Beta variant, they experienced less body weight loss (3% to 5%) after rechallenge of Delta and Omicron variants and no body weight loss in those rechallenged with SARS-CoV-2 prototype. In addition, all of the hamsters showed no significant body weight loss after rechallenge of the same variants (Fig. 2B and Fig. S1). Then, we tested the viral titers in respiratory tract organs, including turbinate (Fig. 2C), trachea (Fig. 2D), and lung (Fig. 2E). After rechallenges of the same or a different variant, infectious viral particles were detectable in turbinate, trachea, and lung in all of the hamsters that were initially infected with Omicron BA.1 variant, whereas initial infection with Beta variant was able to protect hamsters from rechallenge of Beta or prototype SARS-CoV-2. Furthermore, the initial infection of Beta SARS-CoV-2 had a better protection against Delta SARS-CoV-2 rechallenge. Overall, these results suggested that SARS-CoV-2 Omicron BA.1 variant infection resulted in limited protection against rechallenge of other variants in the hamster model. Additionally, in a parallel animal experiment, infectious viral particles were rarely detectable in the respiratory tract organs of Beta and Omicron BA.1 variant from 14 to 25 dpi (Fig. S2).

### Rechallenge of different SARS-CoV-2 variants into hamsters that were initially infected with Omicron BA.1 variant elicited a strong neutralization effect of the serum.

**FIG 3:**
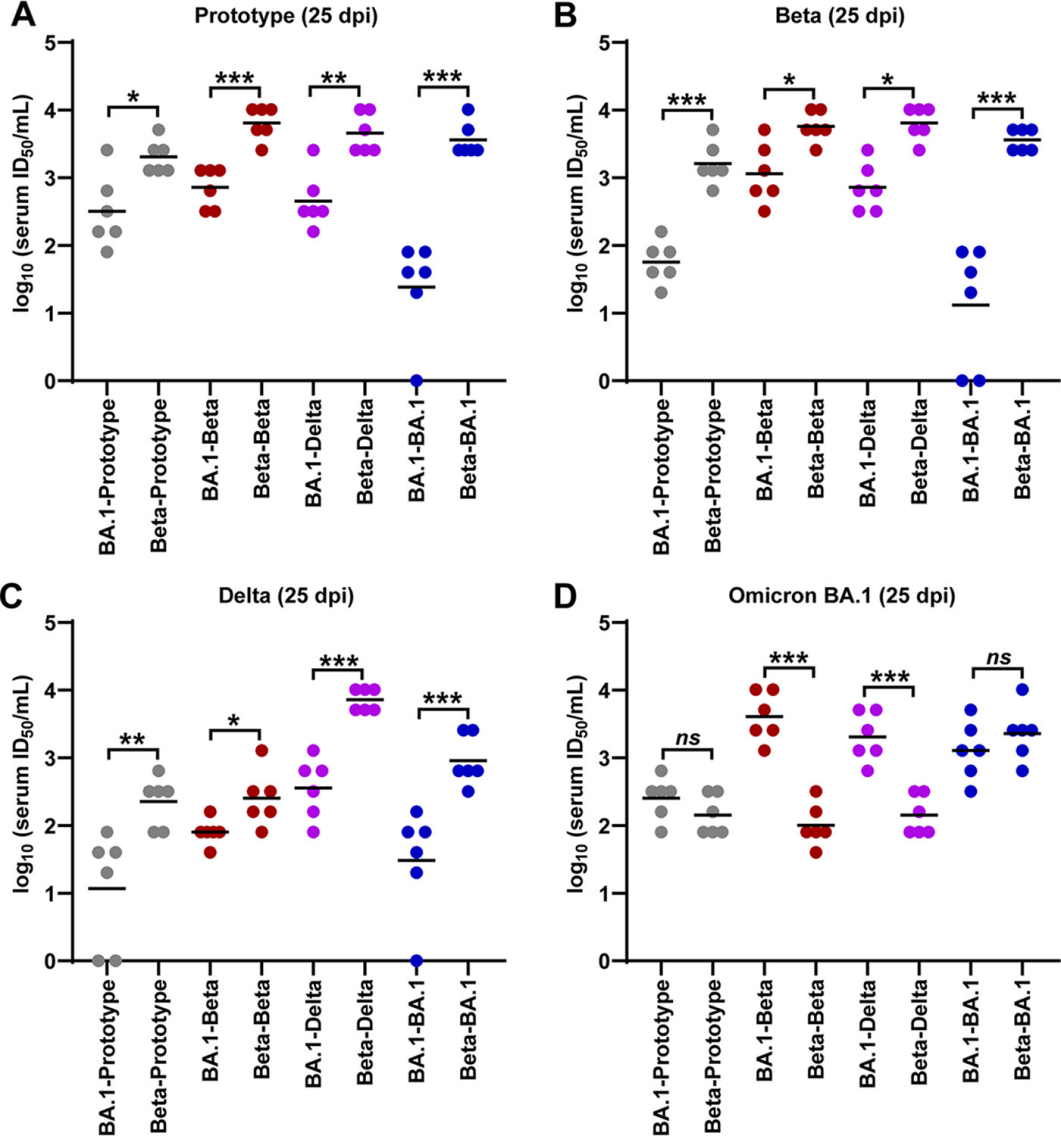
Serological analysis for the hamsters with homologous and heterologous rechallenges. Serum samples were collected at 25 dpi (4 days after rechallenge). The variant-specific serum neutralization titers against SARS-CoV-2 prototype (A), Beta (B), Delta (C), and Omicron (D) BA.1 variants were measured by a by a titration method of TCID_50_ inhibition (*n* = 6/group).

Next, we were curious whether the rechallenges of the same or different SARS-CoV-2 could boost the neuralization effect of the serum. To that end, we collected the serum from the hamsters as shown in Fig. 2A at 25 dpi, and the neutralization assays were performed in Vero cells. As shown in Fig. 3, the serum from the hamsters who were rechallenged with either the same or different SARS-CoV-2 lineages in general presented a better neutralization effect against viral infection in cell culture. In addition, we found that the serum from the hamsters that were initially infected with Omicron BA.1 showed significant lower neutralization titers against SARS-CoV-2 prototype (Fig. 3A), Beta (Fig. 3B), and Delta (Fig. 3C) variants than those from the initial infection of Beta variant. Interestingly, the sequential challenge patterns of BA.1-BA.1, Beta-BA.1, and BA.1-Beta have elicited comparative high serum neutralization titers against Omicron BA.1 variant (Fig. 3D), whereas the sequential challenge pattern of BA.1-prototype cannot elicit high serum neutralization titers against Omicron variant (Fig. 3D). In summary, these data suggested that reinfection with a different variant of SARS-CoV-2 is better than with the same SARS-CoV-2 in inducing serum neutralization titers.

**FIG 4:**
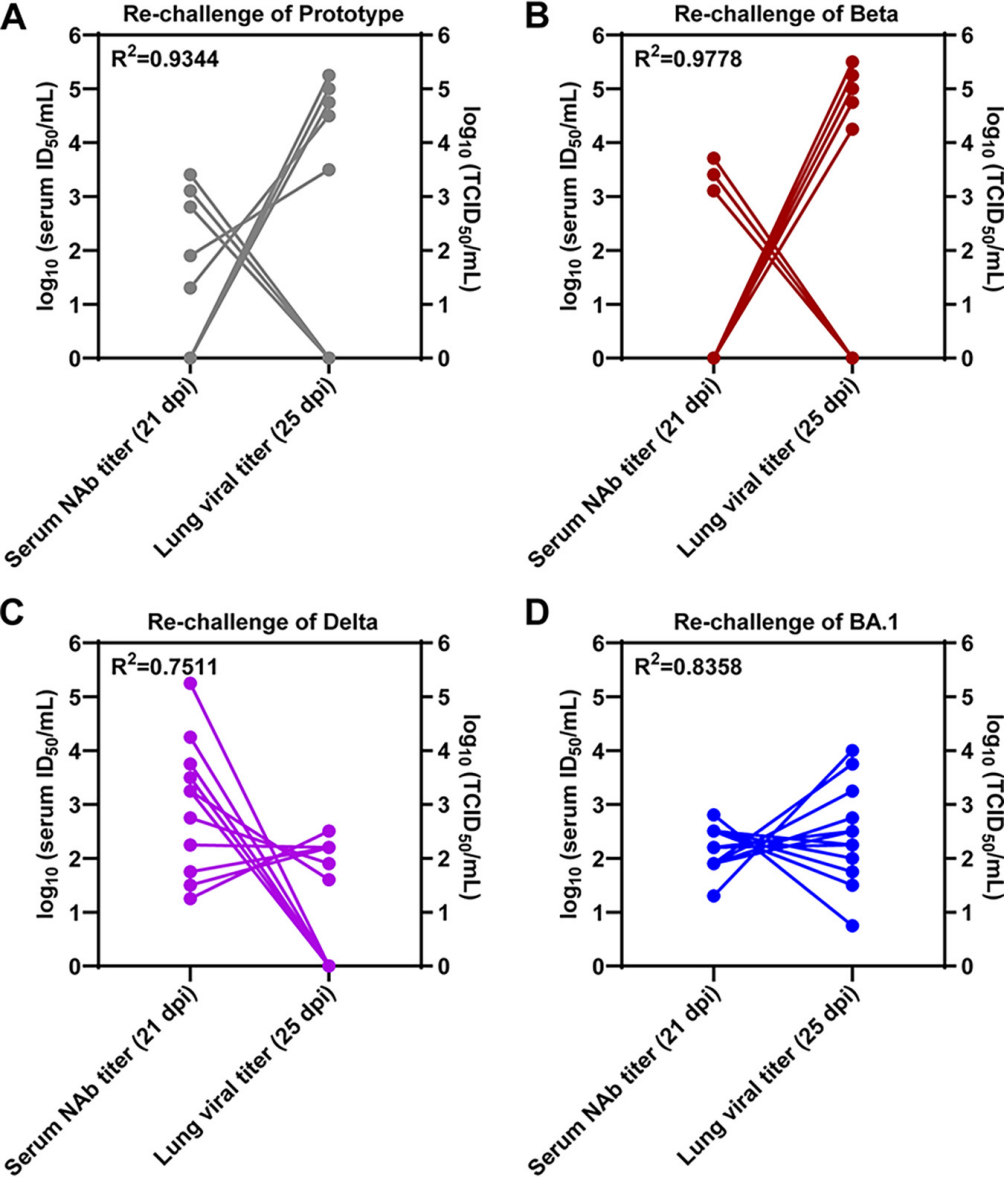
Variant-specific serum neutralization titer indicate the ability to defend homologous and heterologous rechallenge. Hamsters previously exposed to Omicron BA.1 and Beta variants were rechallenged with SARS-CoV-2 prototype (A), Beta (B), Delta (C), and Omicron (D) variants, respectively (*n* = 12/group; 6 hamsters were previously exposed to Omicron BA.1, and 6 hamsters were previously exposed to Beta). In all the groups of homologous and heterologous rechallenge, a high variant-specific serum neutralization antibody (NAb) titer before rechallenges (left *y* axis) suggested low viral titer in lung tissue (right *y* axis) after rechallenges.

In order to determine the neutralization efficacy and viral clearance effects of the serum in a hamster model, we comparatively analyzed the neutralization effect of the serum collected at 21 dpi before reinfection versus viral titers in lung tissues collected at 25 dpi that were 4 days after reinfection (Fig. 2A). The results of correlation analysis among the hamsters previously exposed to Omicron and Beta variants demonstrated that high variant-specific serum neutralization titers against SARS-CoV-2 prototype (Fig. 4A), Beta (Fig. 4B), Delta (Fig. 4C), and Omicron (Fig. 4D) variants before rechallenge indicated low viral titers in lung tissues after rechallenge. For rechallenges of the four different SARS-CoV-2 lineages, 1000 ID50 (50% infective dose) of serum neutralization titer was able to protect against pulmonary virus infection (Fig. 4), whereas the value of more than 100 ID_50_ of serum neutralization titer can largely reduce viral titers in lung (Fig. 4). Furthermore, we analyzed the relationship between variant-specific serum neutralization antibody titers before rechallenge (21 dpi) and viral titers in lung tissues after rechallenge (25 dpi). The results of regression analysis among the hamsters previously exposed to Omicron BA.1 and Beta variants demonstrated that a high variant-specific serum neutralization antibody titer before rechallenge indicated a low infectious virus particle titer in lung tissues after rechallenge (Fig. S3). For rechallenges of the four different SARS-CoV-2 lineages, approximately 1,000 ID_50_ of serum neutralization antibody titer was able to protect against pulmonary virus infection, whereas a value of over 100 ID_50_ of serum neutralization titer can largely reduce viral load in lung tissues.

**FIG 5:**
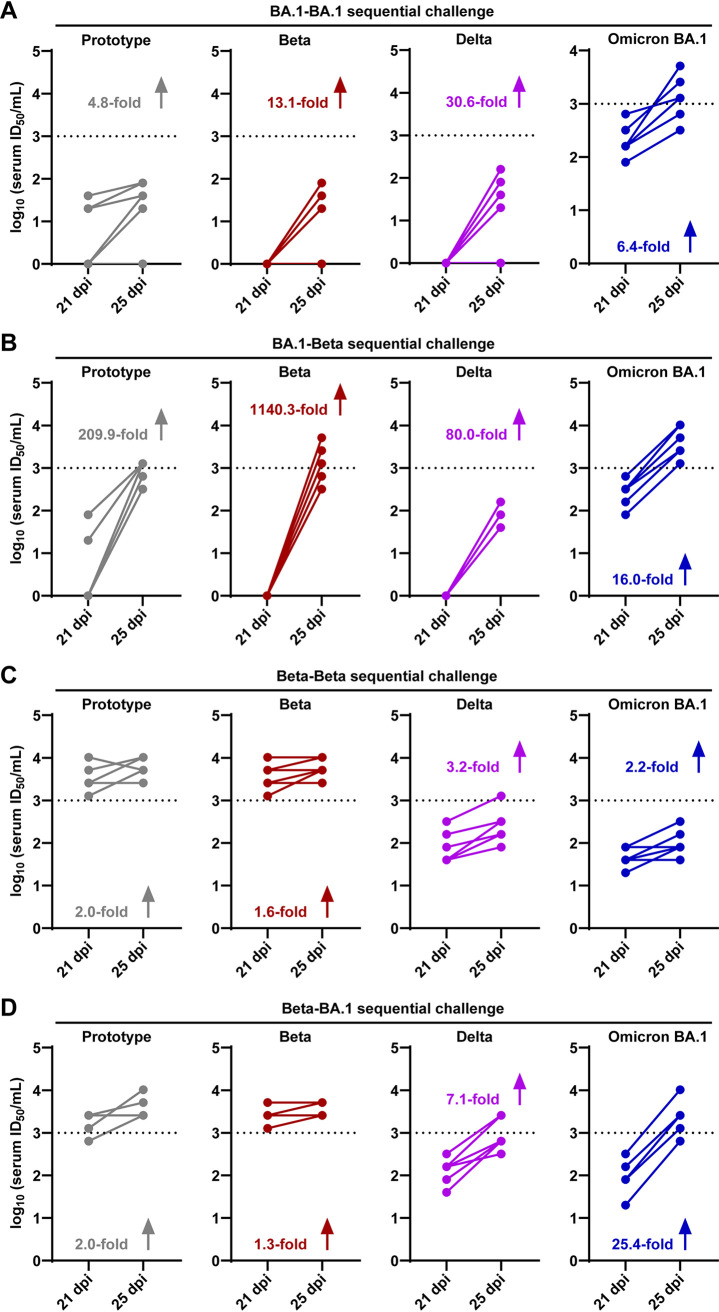
Analysis for the reciprocal changes of variant-specific serum neutralization titers before and after rechallenge. Comparative analysis for the changes in variant-specific serum neutralization titers of the serum samples collected from hamsters with homologous and heterologous sequential infection patterns including Omicron BA.1-Omicron BA.1 (A), Omicron BA.1-Beta (B), Beta-Beta (C), and Beta-Omicron BA.1 (D) were performed (*n* = 6/group). The fold changes of variant-specific serum neutralization titers after rechallenge (25 dpi versus 21 dpi) were calculated.

Of note, the infection and reinfection orders with different SARS-CoV-2 induced different serum neutralization titers, which might lead to a different protection efficiency against a third challenge. Thus, we analyzed the reciprocal changes of variant-specific serum neutralization titers before and after rechallenge in the hamsters with reciprocal sequential challenge patterns of Omicron-Omicron, Beta-Omicron, Beta-Beta and Omicron-Beta, respectively. Generally, the hamsters with Omicron-Omicron (Fig. 5A) or Beta-Beta (Fig. 5C) homologous sequential challenge showed lower increase of variants variant-specific serum neutralization titers than those with Omicron-Beta (Fig. 5B) or Beta-Omicron (Fig. 5D) heterologous sequential challenge. Moreover, rechallenge of Beta variant in the hamsters previously exposed to Omicron variant (Fig. 5B) showed a stronger boost of variant-specific serum neutralization titers than those with a contrary rechallenge pattern (Fig. 5D). Taken together, these results demonstrated that heterologous rechallenge in hamsters previously exposed to Omicron variant elicited a robust increase of variant-specific serum neutralization titers, indicating a strong homologous and cross-variant protection.

## DISCUSSION

The interplay between the evolving SARS-CoV-2 and host immune response has been considered a challenge to design prophylactic vaccines and therapeutic drugs (19–23). After the outbreak of the Omicron variant, its immunogenicity has been described by structural biology (24, 25), screening of neutralizing antibody panels (26–28), and cell-based neutralization assay of previously exposed and vaccinated serum (29–31). Although the tissue tropism and pathogenicity of Omicron variants have been well known (32–35), systematic studies performed in animal models was absence to evaluate the immunogenicity of Omicron variant. A previous study showed that without vaccination, infection with Omicron variant induces a limited humoral immune response in mice and humans (36). However, the immunogenicity changes, and the underlying mechanisms of Omicron infection is not fully understood. To date, a large population has been infected by the same or different lineages of SARS-CoV-2, which necessities the investigation of the immunization effects of serum from a SARS-CoV-2-infected individual against the infection of a different SARS-CoV-2. Therefore, a rechallenge study of the same or different SARS-CoV-2 lineages in a susceptible animal model was urgently needed.

In addition to mice and nonhuman primates, Syrian hamsters have been well demonstrated as a feasible animal model for the study of highly pathogenic coronaviruses (37–39). In this study, we experimentally examined the neutralization effects of serum samples collected from SARS-CoV-2 Omicron BA.1 variant-infected hamsters on different SARS-CoV-2 variants. Moreover, we performed protection experiments to analyze whether an initial infection of Omicron BA.1 or Beta SARS-CoV-2 could protect the hamsters from a reinfection of the same or a different SARS-CoV-2 including the prototype, Beta, Delta, and Omicron BA.1. The initial infection of Omicron BA.1 SARS-CoV-2 resulted in a rapid attenuation of serum neutralization titers from 14 to 21 dpi and loss of the ability to protect a reinfection. In the hamster model, the insufficient heterologous protection against Delta and Omicron BA.1 variants in the hamsters previously exposed to Beta variant was largely derived from the diverse of immunogenicity. The relevance between the variant-specific serum neutralization titers before rechallenge and viral titers in respiratory tract organs after reinfection confirmed that the distinct immunogenicity of Omicron BA.1 and Delta variants might enable escape from the neutralizing antibody elicited by SARS-CoV-2 prototype and Beta variant and cause breakthrough infection. It is important to maintain a high titer of variant-specific neutralization antibody (more than 1,000 ID_50_) so as to protect against SARS-CoV-2 infection and reinfection. Therefore, monitoring the long-term dynamic changes of variant-specific serum neutralization antibody after SARS-CoV-2 infection is necessary to defend a new infection.

Furthermore, the study of rechallenge improves our fundamental understandings of SARS-CoV-2 immunogenicity. The rechallenge of Omicron BA.1 SARS-CoV-2 can effectively boost serum neutralization antibody against itself rather than Prototype, Beta and Delta variants. However, initial infection of Omicron BA.1 and rechallenge with Beta SARS-CoV-2 can boost robust cross-variant serum neutralization antibodies against different SARS-CoV-2 including prototype, Beta, Delta, and Omicron BA.1 variants. Based on these findings, we hypothesize that a vaccination strategy using the spike protein antigens from different SARS-CoV-2 variants might achieve broad protection against the circulating variants. Unfortunately, the Omicron variant caused breakthrough infections among the populations with at least two doses of mRNA or inactivated vaccines that were based on the prototype and a homologous booster (14, 40, 41). In the past months, several new members of the SARS-CoV-2 Omicron variant occupied the dominant position of BA.1 and showed increasing immune escape ability. For instance, SARS-CoV-2 Omicron BA.2, BA.4, and BA.5 lineages caused millions of infection case worldwide with an unexpected mutation rate to escape the preexisting adaptive immunity that derived from BA.1 infection (42–44). Similar to BA.1, the new Omicron variants showed drastic epitope changes and functional shift within the spike protein, attenuated infection, and reduced pathogenicity in lung (33, 45–47), which might contribute to their poor immunogenicity. In addition, further investigation is needed to clarify the underlying reasons for the low protective immunity mounted by Omicron infection. Therefore, development of multivalent SARS-CoV-2 vaccine and optimization of the heterologous vaccination strategy are two promising approaches to enhance our herd immunity to defend the evolving SARS-CoV-2 variants. In conclusion, our study delineated the distinct immunogenicity of the Omicron BA.1 variant and highlighted that heterologous exposure and vaccination is critical to elicit broad-spectrum cross-variant serum neutralization antibodies to combat the emerging and re-emerging SARS-CoV-2 variants.

## MATERIALS AND METHODS

### Experimental animal and biosafety.

The Syrian hamsters were raised in specific pathogen-free animal feeding facilities. All the animal experiments were approved by the Medical Ethics Committee of State Key Laboratory of Emerging Infectious Diseases, School of Public Health, Li Ka Shing Faculty of Medicine, University of Hong Kong and Guangdong-Hong Kong Joint Laboratory of Emerging Infectious Diseases (SUMC2021-112). All experiments with infectious SARS-CoV-2 were performed in biosafety level 3 (BSL-3) and animal biosafety level 3 (ABSL-3) facilities. Our staff wore powered air-purifying respirators that filtered the air and disposable coveralls when they cultured the virus and handled animals that were in isolators. The researchers were disinfected before they left the room and then showered on exiting the facility. All facilities, procedures, training records, safety drills, and inventory records were subject to periodic inspections and ongoing oversight by the institutional biosafety officers who consult frequently with the facility managers.

### Virus stock.

The SARS-CoV-2 prototype (EPI_ISL_1655937), Beta variant (EPI_ISL_2779638), Delta variant (share an identical sequence with EPI_ISL_2385091), and Omicron BA.1 variant (share an identical sequence with EPI_ISL_8182026) were passaged on Vero cells (number CCL-81, ATCC). Viral stocks were prepared in Vero cells with Dulbecco’s modified Eagle’s medium (DMEM) (number 11995) containing 2% fetal bovine (FBS) (number 10270106), 5 μg/mL tosylsulfonyl phenylalanyl chloromethyl ketone (TPCK)-trypsin (number T1426), penicillin (100U/mL)-streptomycin (100 μg/mL) (number 15140-122), and 30 mmol/L MgCl_2_ (these reagents were purchased from GIBCO, Sigma-Aldrich, and Invitrogen). The viruses were harvested and stored in ultralow temperature refrigerator. The titers were determined by means of plaque assay in Vero cells.

### Virus inoculation and sample collection.

Six- to 8-week-old male hamsters were anesthetized by isoflurane (number R510-22, RWD Life Science) and nasally inoculated with the indicated doses of SARS-CoV-2 diluted in 200 μL of phosphate-buffered saline (PBS) (number 10010031, GIBCO). The body weights of these hamsters were measured by an electronic balance. The hamsters were euthanized at the indicated time point for collection of serum and detection of viral load in respiratory tract organs. In this study, we collected 1 g of turbinate and 0.1 g of trachea and lung tissues for detection of viral titer. Because the volume, density, and weight of turbinate, trachea, and lung are quite different, we did not present the results of TCID_50_ as TCID_50_/unit mass of tissue.

### Detection of viral RNA and viral titer.

Viral RNA was extracted by using a QIAamp Viral RNA minikit (number 52906, Qiagen) according to the manufacturer’s instructions. The reverse transcription (RT)-PCR was conducted by using the SLAN-96S real-time system (Hongshi, Shanghai, China) with a SARS-CoV-2 RT-PCR kit from Wantai (WS-1248, Beijing, China). Relative viral RNA of the SARS-CoV-2 ORF1ab gene was determined using primer pairs and probes provided in the kit. Viral RNA copies were expressed on a log_10_ scale after normalized to the standard curve obtained by using 10-fold dilutions of a SARS-CoV-2 stock. The titers of homogenized tissues were measured by plaque assay and half tissue culture infective dose (TCID_50_) titration method in Vero cells seeded in 96-well plates. In the TCID_50_ titration assay, Vero cells were incubated with 100 μL of original tissue homogenates and 10-fold serial diluted samples for 1 h. Then, we renewed fresh medium and observed CPE at 3 days after incubation. We defined that all cells without cytopathic effect indicate “zero.” The serum neutralization titers were measured by a titration method based on TCID_50_ inhibition. We added 10 μL of serum sample in 90 μL of medium for each well and performed 2-fold gradient dilution. After that, 100 μL of serum sample and 100 TCID_50_ virus in 100 μL medium were coincubated with Vero cells for 1 h. Finally, we renewed fresh medium and observed inhibition of CPE at 3 days after coincubation.

### Statistical analysis.

Student’s unpaired two-tailed *t* test and one-way analysis of variance (ANOVA) were performed using GraphPad Prism 8.0 (GraphPad Software). The data are presented as the means ± standard deviation (SD). Two-sided *P* values less than 0.05 were considered significant: *, *P* < 0.01; **, *P* < 0.001; ***, *P* < 0.0001; ns indicates no significance.
