# Supplementary material for: A Heterologous Challenge Rescues the Attenuated Immunogenicity of SARS-CoV-2 Omicron BA.1 Variant in Syrian Hamster Model
Source: J Virol. 2023 Jan 18;97(2):e01684-22. doi: 10.1128/jvi.01684-22 (PMC9972947; doi:10.1128/jvi.01684-22)
Supplement: Supplementary file 1 — Fig. S1 to S3. Download jvi.01684-22-s0001.pdf, PDF file, 0.4 MB [file jvi.01684-22-s0001.pdf]

Supplementary Materials

A heterologous challenge rescues the attenuated immunogenicity of SARS-CoV-2 Omicron BA.1 variant in Syrian hamster model

Jian Ma[1#], Xuan Liu[1#], Ming Zhou[1#], Peiwen Chen[2,3,4#], Rirong Chen[2,3,4], Jia Wang[2,3,4], Huachen Zhu[2,3,4], Kun Wu[1], Jianghui Ye[1], Yali Zhang[1], Quan Yuan[1], Qiyi Tang[5], Lunzhi Yuan[1], Tong Cheng[1], Yi Guan[2,3,4], Ningshao Xia[1]

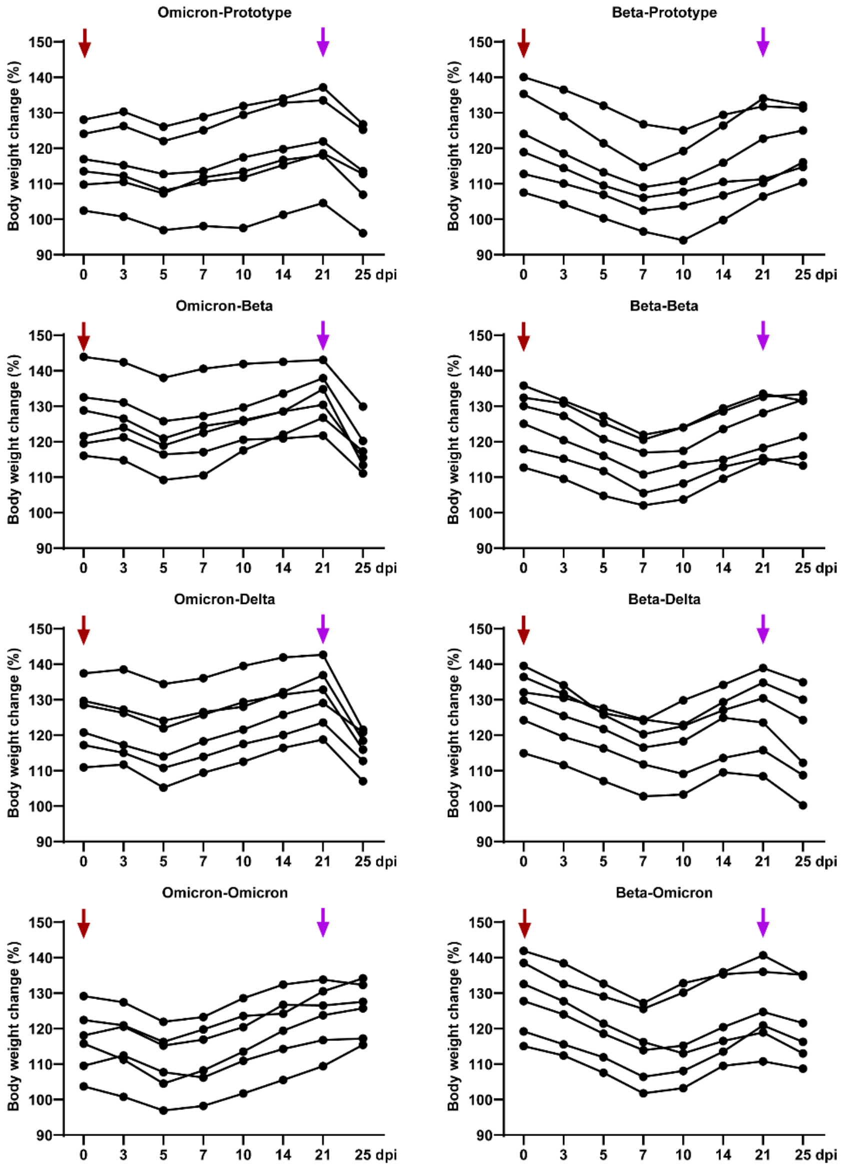

Figure S1. Body weight changes of hamsters from 0 to 25 dpi were recorded. The hamsters were initially infected with Beta or Omicron BA.1 variant at 0 dpi (red arrow), and rechallenged with prototype, Beta, Delta and BA.1 at 21 dpi (purple arrow), respectively.

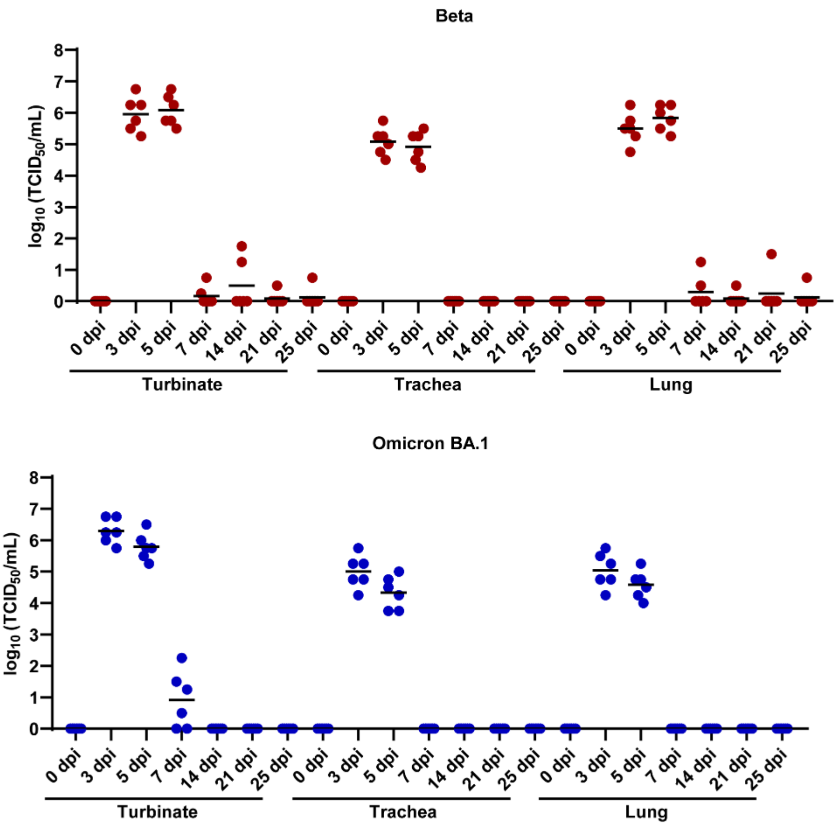

Figure S2. Hamsters were infected with Beta or Omicron BA.1 variant and euthanized at indicated time points for sample collection. Viral titers in the tissues collected from respiratory tract organs including turbinate, trachea and lung were measured by a titration method of $TCID_{50}$. Because the detection limitation of titration method is at least 10 $TCID_{50}$/mL, the undetectable samples are shown as “0” in the figures.

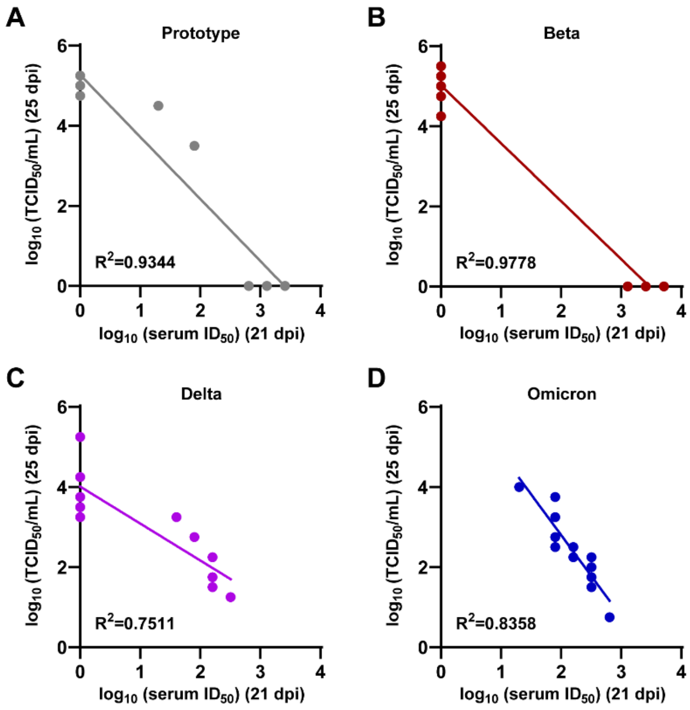

Figure S3. Hamsters prior exposed to Omicron BA.1 or Beta variants were rechallenged with SARS-CoV-2 **(A)** prototype, **(B)** Beta, **(C)** Delta and **(D)** Omicron BA.1 variants, respectively (n=12/group). Regression analysis for the relationship between variant-specific serum neutralization antibody titers at 21 dpi (before rechallenge) and viral titers in lung tissues at 25 dpi (after rechallenge) were performed.
